# Distinct impacts of sodium channel blockers on the strength–duration properties of human motor cortex neurons

**DOI:** 10.1111/epi.18540

**Published:** 2025-07-07

**Authors:** Lorenzo Rocchi, Kate Brown, Alessandro Di Santo, Hannah Smith, Angel V. Peterchev, John C. Rothwell, Ricci Hannah

**Affiliations:** ^1^ University College London London UK; ^2^ University of Cagliari Cagliari Italy; ^3^ University of Waterloo Waterloo Ontario Canada; ^4^ Campus Bio‐Medico University of Rome Rome Italy; ^5^ Duke University Durham North Carolina USA; ^6^ King's College London London UK

**Keywords:** antiseizure medication, axonal membrane, cortex, ion channel, transcranial magnetic stimulation

## Abstract

**Objective:**

This study was undertaken to determine how voltage‐gated sodium channel (VGSC) blockers modulate cortical excitability in vivo. VGSCs are critical for regulating axonal excitability, yet the effects of sodium channel‐blocking medications on human cortical neurons remain poorly characterized. We aimed to address this gap using transcranial magnetic stimulation (TMS)‐derived strength–duration measures as a noninvasive index of VGSC function.

**Methods:**

Thirteen healthy adults received single doses of either carbamazepine, lacosamide, or placebo in a crossover design. TMS was used to assess changes in resting motor threshold and strength–duration properties, including rheobase and the strength–duration time constant, as indices of VGSC function.

**Results:**

Both medications elevated resting motor thresholds compared to placebo, indicating reduced excitability; however, their impacts varied according to TMS pulse width. Carbamazepine raised thresholds proportionally across all pulse widths, whereas lacosamide disproportionately influenced thresholds. Crucially, lacosamide reduced the strength–duration time constant and increased rheobase, whereas carbamazepine had minimal effects on both measures.

**Significance:**

These results reveal subtle and unexpected differences in cortical neuron behavior following VGSC‐blocking medication administration. Lacosamide's response aligns with the proposed mechanism of sodium conductance blockade, whereas carbamazepine's effects suggest distinct VGSC interactions or potential off‐target effects. Our findings advance the understanding of VGSC‐blocking medication interactions in the human cortex and underscore the importance of employing specific TMS measures to gain deeper insights into medication mechanisms of action in vivo. Such measures could serve as valuable adjuncts in medication development and patient monitoring.

**Key points**

- Sodium channel blockers modulate neuronal excitability, but their effects on the human cortex are not fully understood.
- We used novel TMS techniques to investigate how two antiseizure medications, carbamazepine and lacosamide, affect axonal excitability in cortical neurons.
- Both medications elevated resting motor thresholds, indicating reduced net neuronal excitability, but new measures pointed to subtle differences in their effects on sodium channels in the axonal membrane.
- These findings underscore the importance of employing more specific TMS measures to better understand how antiseizure medications influence axonal excitability and neuronal function.

## 1 INTRODUCTION

Voltage‐gated sodium channels (VGSCs) are critical for neuronal electrical signaling and regulation of axonal excitability, with widespread expression in the brain and peripheral nervous system.^1^, ^2^ Rare genetic mutations affecting VGSC function can lead to seizure disorders,^3^ underscoring their importance in the cerebral cortex. Accordingly, sodium channels blockers are a first‐line treatment in some forms of epilepsy.^4^ However, despite their clinical effectiveness and well‐documented impact on neuronal excitability and firing characteristics in animal models and in vitro (for reviews, see Bialer & White^5^ and Rogawski et al.^6^), their in vivo effects on the human brain remain poorly understood due to the lack of noninvasive tools for assessing ion channel function. This knowledge is vital for understanding therapeutic mechanisms and addressing interindividual variability in medication efficacy.^4^

Transcranial magnetic stimulation (TMS) is a noninvasive technique for assessing human cortical excitability. A magnetic pulse applied to the primary motor cortex activates excitatory synaptic inputs to corticospinal neurons, eliciting a muscle twitch on the opposite side of the body.^7^, ^8^ This twitch is quantified via electromyography as the motor evoked potential (MEP). MEPs are used to evaluate motor threshold—the minimum TMS intensity required to produce an MEP of a specific amplitude—or input–output (I‐O) curves, which describe how MEP amplitude changes with varying stimulus intensities.^9^ Previous TMS studies have shown that sodium channel blockers like carbamazepine and lacosamide increase motor thresholds and cause a rightward shift in I‐O curves.^10^, ^11^, ^12^ These changes were assumed to be caused by the blockade of VGSCs in the neuronal membrane.

However, interpreting the results of medication interventions can be complex. First, different medications with distinct mechanisms of action can lead to the same outcome. Medications that interfere with synaptic transmission at excitatory and inhibitory synapses can also produce changes in motor thresholds.^13^, ^14^, ^15^, ^16^ Second, certain medications may have multiple actions. For instance, carbamazepine may affect γ‐aminobutyric acid (GABA) receptor conductance, calcium channel function, and serotonin release.^17^, ^18^, ^19^ Hence, single motor threshold measurements lack specificity for identifying changes in axonal excitability, often necessitating complementary measures of intracortical excitation and inhibition, presumed to examine synaptic transmission,^20^ to rule out “off‐target” effects.

Here, we sought to confirm the sodium channel‐blocking action using a complementary measure of sodium channel function: the strength–duration time constant (SDTC), which reflects how threshold varies with stimulus strength (amplitude) and duration (width).^21^ The SDTC is thought to reflect the dynamics of VGSCs,^22^, ^23^, ^24^ and prior studies in humans and rodents have shown that sodium channel blockers like lacosamide,^25^ mexiletine,^26^ and ranolazine^27^ reduce SDTC in peripheral nerves. Our goal was to determine whether neurons in the cortex exhibit similar responses, elucidating potential changes in axonal excitability driven by sodium channel blockade.

We used a TMS device capable of varying pulse width,^28^ which enables measurement of the SDTC of motor cortical neurons,^29^, ^30^, ^31^, ^32^ to investigate the effects of single doses of carbamazepine and lacosamide in healthy individuals. These medications were chosen due to their distinct effects on sodium channel function and neuronal firing, as evidenced in animal and in vitro experiments.^6^ We tentatively predicted they would produce divergent effects on the strength–duration behavior. Despite differences in sodium channel subtype distribution between the cortex and periphery,^33^ most sodium channel blockers act nonselectively. We therefore hypothesized that cortical effects would align with those previously observed in peripheral nerves.

## 2 MATERIALS AND METHODS

### 2.1 Participants

Thirteen healthy, young adults (mean age = 25 ± 5 years, 7 females) volunteered, providing written informed consent. The sample size is consistent with earlier studies demonstrating effects of lacosamide and carbamazepine on cortical excitability.^10^, ^11^, ^12^ Procedures adhered to the Declaration of Helsinki and were approved by the University College London Research Ethics Committee (Project ID: 5732/003). All participants were free from neurological or psychiatric disorders and not taking neuroactive medications.

### 2.2 Experimental design

This placebo‐controlled, single‐blind, repeated‐measures study involved three laboratory visits, during which participants—blinded to treatment condition—received carbamazepine, lacosamide, or placebo in a randomized order. Washout periods lasted at least 7 days. TMS measurements were conducted at a single time point corresponding to the presumed peak plasma concentration of the medications: 6 h post carbamazepine^34^, ^35^ and 1 h post lacosamide^36^ or placebo. Although the absolute timing differed between drugs, this approach ensured that each condition was assessed at its expected peak pharmacological effect, thereby supporting valid comparisons of their presumed peak impact on cortical excitability. Measurements included a preliminary estimate of resting motor threshold (RMT) and I‐O curves.

### 2.3 Medications

The placebo was a lime and mint‐flavored drink, also used to mask the taste of the antiseizure medications. Carbamazepine (600 mg) and lacosamide (200 mg) were administered in liquid form, with doses selected based on prior evidence of their effects on motor thresholds and I‐O curves^10^, ^11^, ^12^ and peripheral nerve SDTC.^25^

### 2.4 Electromyography

Electromyographic signals were recorded using a bipolar muscle–tendon montage with one electrode positioned over the belly of the right first dorsal interosseous muscle and the reference over the second metacarpophalangeal joint, with the ground electrode placed on the ulnar styloid process. Signals were amplified (×1000; Digitimer D360, Digitimer) and bandpass filtered (5–3000 Hz). They were digitized at 5000 Hz (Power1401, Cambridge Electronic Design) and analyzed with Signal software version 5.10 (Cambridge Electronic Design).

### 2.5 Transcranial magnetic stimulation

**FIGURE 1:**
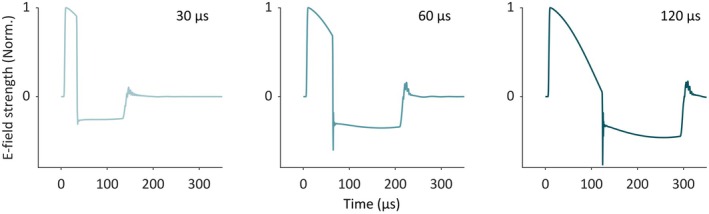
Electric field waveforms recorded with a search coil for each of the three pulse widths of the initial phase (30, 60, and 120 μs). Amplitudes are normalized to unity. Maximum stimulator output was limited to 100%, 73%, and 50% for each of the pulse widths, respectively.

TMS was delivered to the left motor cortex via a device connected to a 70‐mm figure‐of‐8 coil (Elevate TMS, Rogue Research) to evoke MEPs in the right first dorsal interosseous muscle. The induced electric field waveform (Figure 1) had a pseudorectangular shape with an M‐ratio of .2 (ratio of the amplitude of the initial phase to that of the second phase), favoring unidirectional electric field pulses. We used three different pulse widths (Figure 1).

### 2.6 Experimental protocol

Participants received the medication or placebo and waited/returned to the lab 1 h (placebo, lacosamide) or 6 h (carbamazepine) before TMS procedures. Participants were then seated. The TMS coil was positioned over the left M1 with the handle angled ~45° posterolaterally to induce posterior–anterior initial currents with respect to motor cortex. The motor hotspot was identified by locating the position where slightly suprathreshold 120‐μs pulses elicited the largest and most consistent MEPs in the first dorsal interosseous muscle, and marked on the participant's cap.

**FIGURE 2:**
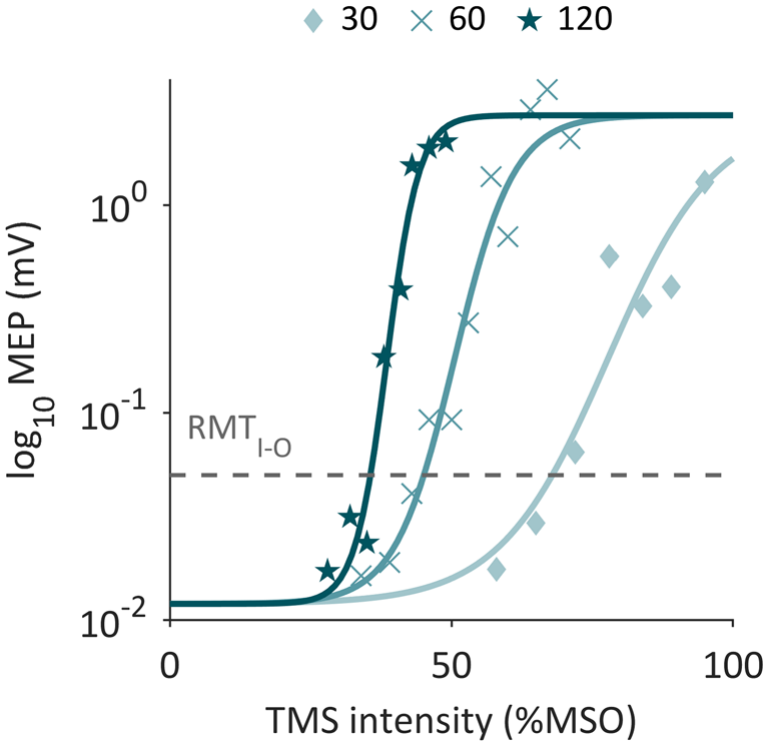
Example input–output (I‐O) curves for each pulse width from a single participant. Median log‐transformed motor evoked potential (MEP) peak‐to‐peak amplitude is shown as a function of transcranial magnetic stimulation (TMS) stimulus intensity (percentage of maximum stimulator output [%MSO]). The curves were used to estimate resting motor threshold (RMT_I‐O_). The group average model fits, based on the Boltzmann sigmoidal curve, exhibited *R* ^2^ values ranging .94–.97 across all pulse widths and conditions. Individual I‐O curves for each session are provided in Figures S1–S3 (available at https://osf.io/d6vf2/). Note that stimuli were delivered at intensities corresponding to 80%, 90%, 100%, 108%, 116%, 124%, 132%, 140%, 148%, 156%, and 164% of each participant's preliminary RMT.

Preliminary RMT estimates for each pulse width were determined at the hotspot by finding the intensity that evoked MEPs > .05 mV in 5 of 10 trials. These values guided stimulus intensities for I‐O curves, which provided more robust RMT estimates. Ten pulses were delivered at 11 intensities for each pulse width (Figure 2). Within each pulse width, the order of stimulus intensities was randomized.

### 2.7 Data analysis

The strength–duration behavior of nodal membranes can be described by two parameters: the SDTC, describing the relationship between the strength (amplitude) and duration (width) of a pulse needed to elicit an action potential; and the rheobase, the extrapolated threshold for a pulse of infinite duration.^22^, ^37^ As in previous TMS studies, we employed the RMT to characterize the strength–duration behavior.^30^, ^32^

#### 2.7.1 Estimating RMTs from I‐O curves

We used the I‐O curve to derive a more precise RMT estimate (RMT_I‐O_). For each pulse width, 10 stimuli were delivered per intensity, with values log‐transformed (log_10_) and the median response at each intensity calculated. These medians were used to fit a sigmoidal curve (see Figure 2):

(1) $$y=y_{l}+\frac{\left(y_{h}-y_{l}\right)}{1+\exp\left(\frac{x_{m}-x}{s}\right)}$$

Here x is the stimulus intensity (% maximum stimulator output), y is the log‐transformed MEP amplitude (mV), $y_{l}$ and $y_{h}$ are the lower and upper saturation levels, $x_{m}$ is the stimulus intensity at the midpoint of the curve (% maximum stimulator output), and *s* is the spread or width of the curve specifically describing the speed of transition between the lower and upper asymptotes.

Saturation levels ($y_{l}$ and $y_{h}$) were fixed across pulse widths within each session. $y_{l}$ was determined as the 10th percentile of the peak‐to‐peak electromyographic signal amplitudes measured in a 35‐ms window prior to TMS, reflecting the background noise. $y_{h}$ was set as the 90th percentile of the peak‐to‐peak MEP amplitudes, measured in a 35‐ms window after the TMS pulse (15–50 ms). Both were calculated across all pulse widths and stimulus intensities within a session. Curve fitting adjusted *s* and $x_{m}$ to optimize the fit of the curve to the data. The parameters from the model were used to estimate RMT_I‐O_ as the stimulus intensity producing an MEP amplitude of .05 mV after log transformation, that is, log_10_(.05). We fitted these curves for each pulse width of each experimental condition and participant.

#### 2.7.2 Strength–duration time constant

We employed similar procedures to those in our previous studies,^31^, ^32^ deriving strength–duration curves for each experimental condition using RMT_I‐O_ across pulse widths. The SDTC models how stimulus intensity changes with pulse width, which depends on the local electric field and axonal membrane properties. This relationship is described by the equation:

(2) $$V_{\mathrm{th}}^{\prime }\left(t_{p}\right)=\frac{V_{\mathrm{th}\infty }}{r\left(\tau_{m},t_{p}\right)}$$

where $V_{\mathrm{th}}^{\prime }$($t_{p}$) is the modeled RMT (the threshold required to evoke a motor response), *V*_*th∞*_ is the rheobase, $t_{p}$ is the pulse width, $\tau_{m}$ is the SDTC, and *r*($\tau_{m}$ $t_{p}$) is a depolarization factor representing how effectively the TMS pulse depolarizes the membrane.^32^

To estimate the rheobase and SDTC, we fitted the parametric model $V_{\mathrm{th}}^{\prime }$($t_{p}$) to the experimental RMT_I‐O_ data, $V_{\mathrm{th}}$($t_{p}$). We did this by minimizing the difference between the predicted and actual RMT_I‐O_ values using the following least‐squares method:

(3) $$\sum {\left(\frac{V_{\mathrm{th}}^{\prime }\left(t_{p}\right)\;}{V_{\mathrm{th}}\left(t_{p}\right)\;}-1\right)}^{2}$$

This method helps to find the best‐fitting values for rheobase and SDTC by reducing discrepancies between the model and the experimental data. The SDTC and rheobase calculations were performed in MATLAB (R2024b, MathWorks). Parameters were estimated for each participant and each experimental condition under the assumption of individual SDTC and rheobase values.

### 2.8 Statistical analyses

Data are presented as mean and SEM. To examine the effects of medication condition and pulse width on RMT_I‐O_, we used linear mixed‐effects models, with analysis of variance (ANOVA) applied to the fixed effects. This approach accounts for interindividual variability by including subject‐specific random intercepts and slopes, thereby modeling the correlation structure of repeated measures within subjects. The fixed effects tested included medication condition (placebo, carbamazepine, and lacosamide), pulse width (30, 60, and 120 μs), and their interaction.

To examine the effects of medication condition on SDTC and rheobase, we used a similar linear mixed effects model approach, including random intercepts to account for subject‐specific variability. The placebo condition was treated as the reference category, and the effects of carbamazepine and lacosamide were estimated relative to this reference. SDTC and rheobase are linearly and inversely related,^32^, ^38^, ^39^ likely due to their shared dependence on sodium conductance. We therefore expected the medications to exert opposing effects on these indices. Linear regression evaluated the relationship between SDTC and rheobase for each condition. Additionally, a linear mixed effects model examined this relationship across conditions, including medication condition and their interaction as fixed effects, with random intercepts to account for within‐participant variability.

All statistical analyses were conducted with a significance level set at *p* < .05. Effect sizes for fixed effects are reported using Cohen *d*, calculated as the fixed effects estimate divided by the residual SD.

## 3 RESULTS

### 3.1 Side effects

The medications were generally well tolerated, with only mild side effects including drowsiness (carbamazepine, *n* = 9 participants; lacosamide, *n* = 7 participants), erythema (carbamazepine, *n* = 1), dizziness (carbamazepine, *n* = 1), nausea (carbamazepine, *n* = 1) and mild ataxia (carbamazepine, *n* = 1). None of the side effects interfered with participants' ability to complete the experiments.

### 3.2 Single motor threshold analysis

**FIGURE 3:**
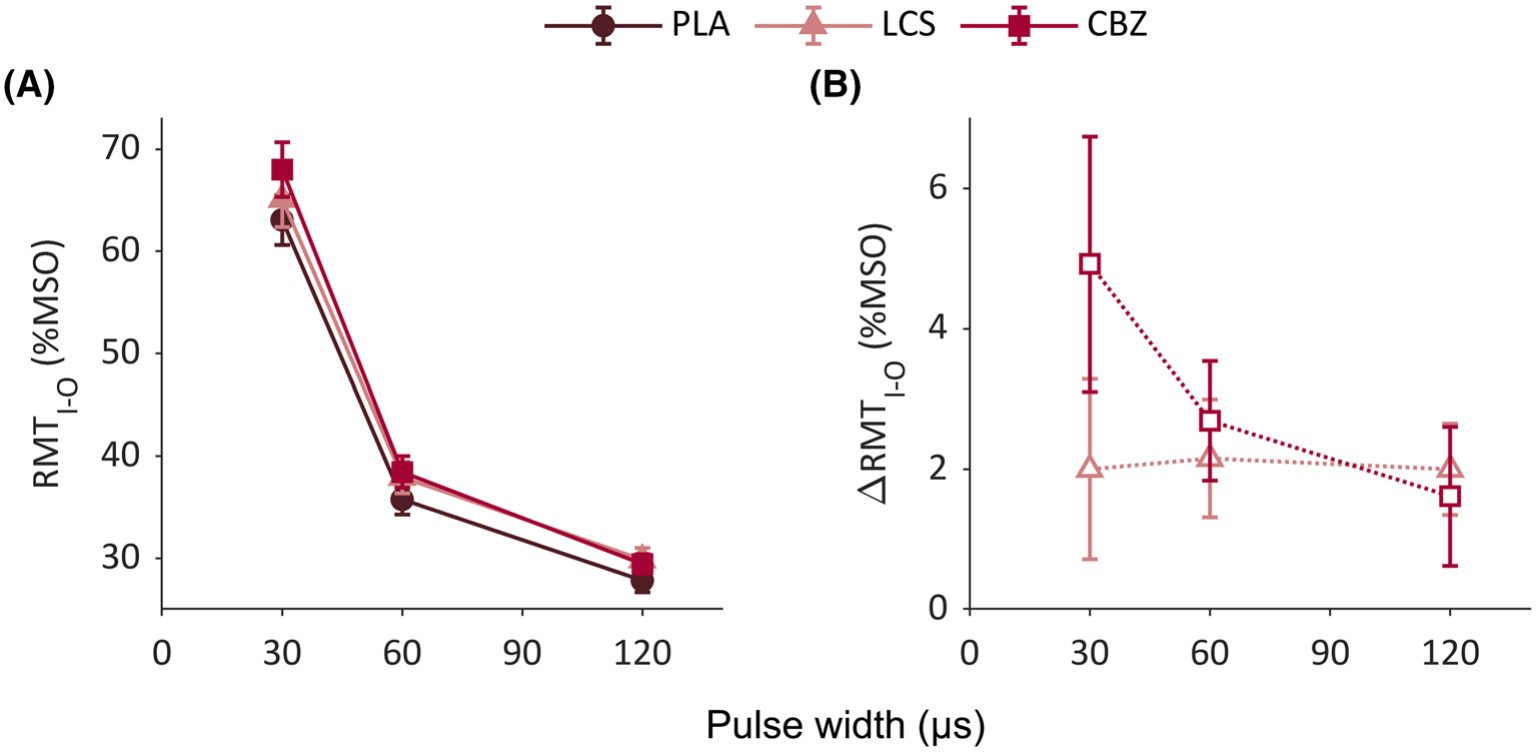
Strength–duration behavior derived from estimates of resting motor threshold obtained using the input‐output curves (RMT_I‐O_). (A) Strength–duration curves of RMT_I‐O_ as a function of pulse width (30, 60, 120 μs) for each condition (placebo [PLA], carbamazepine [CBZ], lacosamide [LCS]). Motor thresholds (expressed as a percentage of maximum stimulator output [%MSO]) decreased with increasing pulse width (main effect of pulse width: *F*_[2,108]_ = 228.75, *p* = 1.49 × 10^−39^), and were elevated under carbamazepine (estimate = 4.92 ± .99, *t*_[108]_ = 4.99, *p* = 2.36 × 10^−6^) and lacosamide (estimate = 2.00 ± .99, *t*_[108]_ = 2.03, *p* = .045) relative to placebo (main effect of medication: *F*_[2,108]_ = 12.58, *p* = 1.23 × 10^−5^). The interaction between pulse width and medication was not significant (*F*_[4,108]_ = 1.97, *p* = .104). (B) Differences in RMT_I‐O_ between each drug and placebo across pulse widths. Positive values indicate an increase relative to placebo. Carbamazepine effects varied with pulse width, producing larger increases for shorter pulses, which have higher absolute thresholds. By contrast, lacosamide produced a consistent increase in motor threshold across all pulse widths. A follow‐up linear mixed effect confirmed a condition × pulse width interaction (*F*_[2,72]_ = 3.35, *p* = .041). Data are mean ± SEM.

We first analyzed the impact of each medication on a single threshold, RMT_I‐O_ measured at 60 μs (Figure 3A), which is close to a standard ~80‐μs pulse as used in previous studies. The analysis showed significant main effects for medication (*F*_[2,36]_ = 6.20, *p* = .005). Fixed effect estimates showed that both carbamazepine (estimate = 6.69, SE = .81, *t*_[36]_ = 3.33, *p* = .002, Cohen *d* = 1.31) and lacosamide (estimate = 2.15, SE = .81, *t*_[36]_ = 2.66, *p* = .012, Cohen *d* = 1.04) significantly increased RMT_I‐O_ compared to placebo. To directly compare the effects of carbamazepine and lacosamide, we ran a similar linear mixed effects model with carbamazepine as the reference category. This model showed no significant difference between the effects of carbamazepine and lacosamide (estimate = −.54, SE = .81, *t*_[36]_ = −.67, *p* = .51, Cohen *d* = −.21). Hence, analysis of single motor thresholds did not distinguish between the effects of the two medications.

### 3.3 Strength–duration curves

We next examined strength–duration behavior using RMT_I‐O_ values derived from I–O curves. Motor thresholds decreased with increasing pulse width and were elevated under both drug conditions compared to placebo (Figure 3A; see legend for omnibus statistics).

To examine medication‐specific effects, we conducted follow‐up linear mixed effect models comparing placebo against each drug and against each other. For lacosamide versus placebo, there was a main effect of condition (*F*_[1,72]_ = 5.58, *p* = .021), with no interaction (*F*_[2,72]_ = .011, *p* = .99), indicating a uniform threshold increase across pulse widths. For carbamazepine versus placebo, there was a main effect of condition (*F*_[1,72]_ = 21.16, *p* = 1.77 × 10^−5^) and a trend toward an interaction (*F*_[2,72]_ = 2.49, *p* = .091), suggesting pulse width‐dependent effects.

A direct comparison between carbamazepine and lacosamide revealed a significant interaction (condition: *F*_[1,72]_ = 9.82, *p* = .003; pulse width: *F*_[2,72]_ = 4.24, *p* = .018; interaction: *F*_[2,72]_ = 4.05, *p* = .021; Figure 3B). Altogether, this indicates that carbamazepine's effect scaled with pulse width, unlike lacosamide.

To visualize this more effectively, we calculated the difference in RMT_I‐O_ between each medication condition and placebo (Figure 3B). Carbamazepine exhibited pulse width‐dependent variations in absolute RMT_I‐O_ differences, with larger shifts at shorter pulse widths where thresholds are higher. Consequently, absolute differences at each pulse width were broadly proportional to the magnitude of RMT_I‐O_. In contrast, lacosamide showed consistent absolute differences that were therefore not proportional to RMT_I‐O_ across pulse widths. This was supported by the ANOVA on the differences within the same linear mixed effects model framework, which showed an interaction (condition: *F*_[1,72]_ = 9.82, *p* = .003; pulse width: *F*_[2,72]_ = 4.24, *p* = .018; interaction: *F*_[2,72]_ = 3.35, *p* = .041).

### 3.4 Strength–duration time constant and rheobase estimates

**FIGURE 4:**
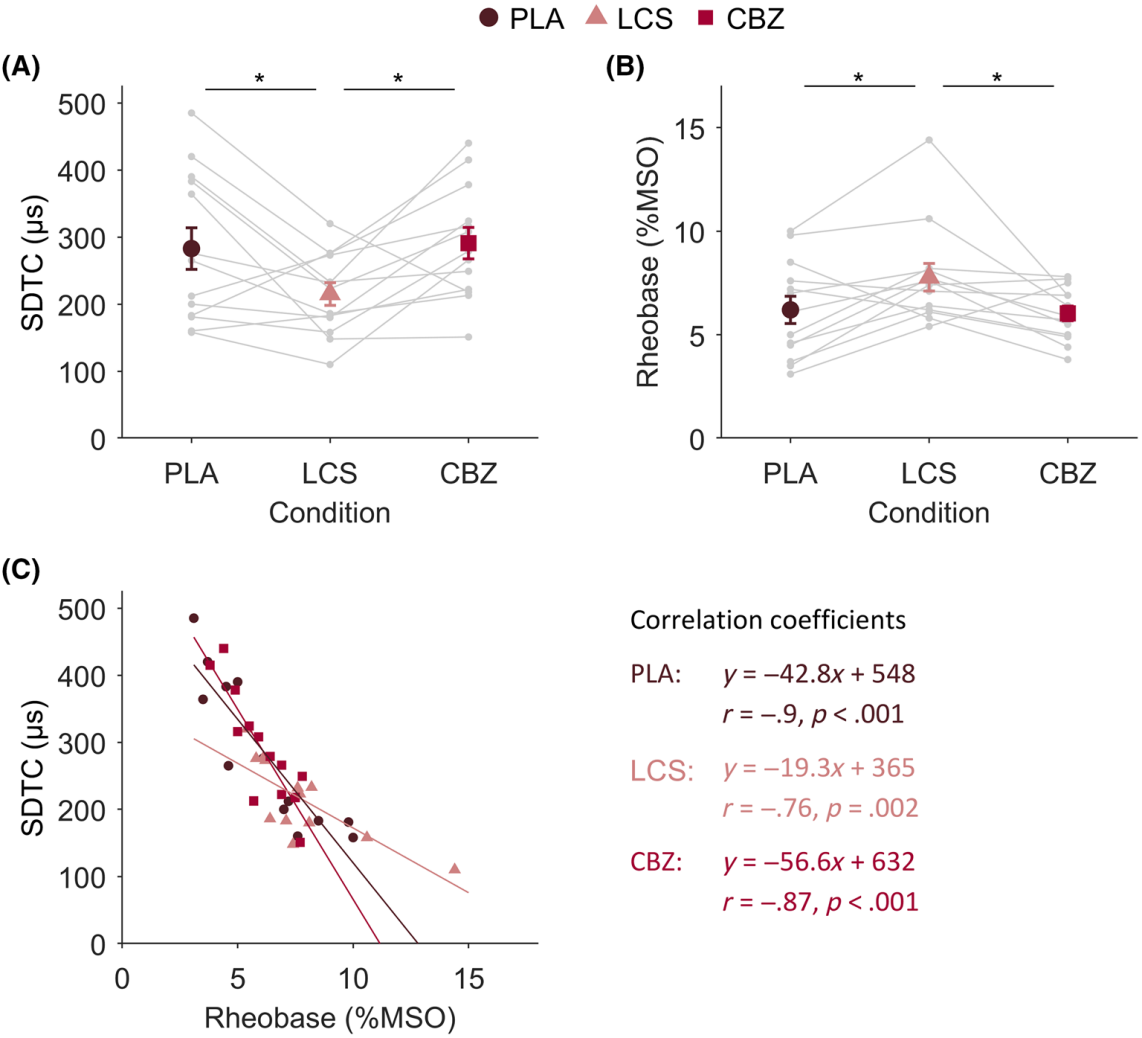
Strength–duration behavior derived from estimates of resting motor threshold obtained using input‐output curves (RMT_I‐O_). (A) Strength–duration time constant (SDTC) estimates. Lacosamide significantly reduced SDTC compared to placebo (estimate = −67.6, SE = 30.4, *t*_[36]_ = −2.22, *p* = .032, Cohen *d* = −.87) and carbamazepine (estimate = −75.5, SE = 30.4, *t*_[36]_ = −2.48, *p* = .018, Cohen *d* = −.97), with no difference between placebo and carbamazepine (estimate = 7.85, SE = 30.4, *t*_[36]_ = .26, *p* = .798, *d* = −.10). Analysis of variance (ANOVA) confirmed a main effect of condition (*F*_[2,36]_ = 3.73, *p* = .034). (B) Rheobase estimates. Lacosamide significantly increased rheobase compared to placebo (estimate = 1.57, SE = .66, *t*_[36]_ = 2.38, *p* = .023, Cohen *d* = .93) and carbamazepine (estimate = 1.57, SE = .66, *t*_[36]_ = 2.38, *p* = .023, *d* = .94), with no difference between placebo and carbamazepine (estimate = −.17, SE = .66, *t*_[36]_ = −.26, *p* = .799, *d* = −.10). ANOVA confirmed a main effect of condition (*F*_[2,36]_ = 4.24, *p* = .022). (C) Relationship between SDTC and rheobase across individuals in each condition. Strong inverse correlations were observed for all conditions (all *p* < .01). Regression slopes varied by condition, with lacosamide showing a less negative relationship. Lines show best‐fitting regressions per condition. Data in panels A and B are mean ± SEM; individual data points are shown as small dots. %MSO, percentage of maximum stimulator output; CBZ, carbamazepine; LCS, lacosamide; PLA, placebo. *, *p* < .05.

We then estimated SDTC and rheobase to formally characterize the strength–duration curves (Figure 4A,B; see legend for main ANOVA and pairwise statistics). Lacosamide consistently reduced SDTC and increased rheobase relative to both placebo and carbamazepine, whereas no differences were observed between placebo and carbamazepine.

To mitigate potential overfitting from estimating SDTC per participant, we repeated the analysis assuming a common SDTC within each condition and individual rheobase values.^32^ This approach confirmed a similar pattern of results (Table S1, available at https://osf.io/d6vf2/).

To further explore the seemingly opposing effects on SDTC and rheobase, we evaluated the relationship between the variables for each medication condition. We found that individual estimates of SDTC and rheobase were strongly and inversely related to one another within each condition (Figure 4C; all *p* < .01).

Moreover, the slope of the relationship appeared to differ across the three conditions, as supported by the results of the linear mixed effects model. The main effect of rheobase indicates a strong negative relationship (estimate = 46, SE = 3.2, *t*_[*df*]_ = −14.0_[33]_, *p* = 1.8 × 10^−15^), where each 1% increase in rheobase corresponds to an approximately 46μs reduction in SDTC. The intercept in the model represents the estimated SDTC for the placebo condition (estimate = 566, SE = 23, *t*_[*df*]_ = 24.2_[33]_, *p* = 1.5 × 10^−22^), with all predictors set to their reference levels, and the estimates for lacosamide and carbamazepine reflect their effect relative to this baseline. Relative to placebo, lacosamide produced a significant reduction in SDTC (estimate = −156, SE = 29, *t*_[*df*]_ = −5.44_[33]_, *p* = 5.1 × 10^−6^) and a shallower slope (estimate = 20.6, SE = 3.9, *t*_[*df*]_ = 5.28_[33]_, *p* = 8.1 × 10^−6^). Carbamazepine showed a trend toward increased SDTC (estimate = 84.2, SE = 41.9, *t*_[*df*]_ = 2_[33]_, *p* = .053) and a more negative slope compared to placebo (estimate = −13.9, SE = 6.8, *t*_[*df*]_ = −2.06_[33]_, *p* = .047). However, the effects were smaller in magnitude than those associated with lacosamide.

## 4 DISCUSSION

We used a novel TMS device to study the effects of sodium channel‐blocking medications on the strength–duration behavior of neurons in the human motor cortex. Both carbamazepine and lacosamide increased motor thresholds, reducing cortical excitability in line with previous studies using a single pulse width.^10^, ^11^, ^12^ However, single‐threshold measurements alone could not distinguish between the effects of the two medications. By employing strength–duration curves, we uncovered distinct pulse width‐dependent effects; carbamazepine increased thresholds proportionally across all pulse widths, whereas lacosamide's effects were proportionally larger at longer duration pulses. These differences were further reflected in the SDTC and rheobase parameters, with lacosamide reducing SDTC, increasing rheobase, and altering their relationship slope, whereas carbamazepine produced only minor and inconsistent effects. Our findings demonstrate the utility of strength–duration metrics in uncovering subtle medication‐specific effects on VGSC function, which may be overlooked by single pulse width approaches.

### 4.1 Motor thresholds

The motor threshold is directly proportional to the magnitude of membrane depolarization of the neurons stimulated by TMS,^32^ which depends on the stimulus strength and duration. However, because TMS activates corticospinal neurons transsynaptically, changes in a single motor threshold may involve synaptic as well as axonal contributions, making it challenging to isolate the cause. Strength–duration curves, derived from thresholds across multiple pulse widths, address this limitation by focusing on axonal membrane changes. As pulse width increases, the degree of membrane depolarization also increases, lowering the motor threshold. This shapes the strength–duration curve, which primarily reflects axonal excitability, as axonal depolarization precedes synaptic activity summation. As such, strength–duration curves offer a more precise measure than a single threshold.

### 4.2 TMS‐derived strength–duration curves as indices of axonal excitability

Recent work suggests that the cortical SDTC is sensitive to the TMS stimulus waveform, potentially reflecting differential polarization of the axonal membrane and variations in sodium conductance.^30^ This reinforces the idea that strength–duration curves are specific to axonal properties and can serve as indicators of VGSC‐mediated changes in cortical axonal excitability. Computational modeling also points to the axon, likely near the terminal, as the site of activation for cortical neurons,^7^, ^40^ further supporting the interpretation that strength–duration curves reflect sodium channel changes at these sites.

### 4.3 Medication‐induced changes in strength–duration behavior

Lacosamide significantly altered strength–duration parameters, increasing rheobase and reducing SDTC, highlighting their potential as biomarkers for medication action. These effects mirror those observed in peripheral nerve SDTC.^25^ Although peripheral changes are plausible, the present findings are more likely to reflect cortical effects. This is because the shape of the strength–duration curve primarily reflects excitability of axons depolarized directly by the TMS pulse, upstream of both cortical synaptic transmission and peripheral motor nerve activation (see Section 4.1). As such, peripheral changes are unlikely to account for the effects observed here.

In contrast, carbamazepine had minimal impact on SDTC or rheobase despite reducing cortical excitability. Notably, preliminary findings from a study indicated that carbamazepine altered the SDTC and rheobase of peripheral sensory nerves in both humans and rodents but not in human motor nerves, despite producing changes in other excitability measures.^41^ Our findings regarding motor cortical axons align with the behavior observed in peripheral motor axons.

The SDTC is influenced by resting sodium conductance, membrane potential, and passive axonal properties like myelination.^21^, ^22^, ^23^ Previous studies of human peripheral nerve excitability after short‐term administration with lacosamide^25^ and long‐term administration of mexiletine^26^ attributed SDTC increases to changes in sodium conductance. Further evidence comes from cases of accidental tetrodotoxin ingestion, where the potent sodium channel blocker reduced the SDTC.^42^ Modeling of the toxin‐induced changes in axonal excitability supported VGSC blockade as the likely cause. We therefore interpret our results as being consistent with the proposed VGSC‐blocking action of lacosamide.

Membrane potential shifts could also theoretically explain lacosamide‐induced changes in strength–duration behavior.^39^, ^43^ However, as noted previously,^44^ the low slopes of the relationships between membrane potential and both SDTC and rheobase^43^, ^45^ suggest that a large hyperpolarizing shift would be needed to explain changes in strength–duration behavior.

The differential effects of carbamazepine and lacosamide suggest distinct mechanisms of action, although we can only speculate on the specifics. Axonal membranes contain VGSCs that conduct transient and persistent sodium currents, both of which influence the SDTC, although the latter is more commonly associated with it.^22^, ^23^, ^30^, ^46^ In vitro experiments show that carbamazepine and lacosamide reduce both transient and persistent sodium currents^47^, ^48^, ^49^, ^50^ (reviewed in Rogawski et al.^6^). However, work in mice has shown that carbamazepine induces a hyperpolarizing shift in the activation of persistent sodium currents.^50^ This produced a significant increase in persistent sodium current conductance at subthreshold voltages in *SCN1B* knockout mice, which lack the β1 subunit, but not in wild‐type mice. This genotype‐specific effect could, in some cases depending on β subunit composition, reduce the drug's effectiveness at suppressing the persistent currents that influence the SDTC. Another relevant factor is that motor axons are known to have lower SDTCs than sensory axons, potentially reflecting reduced persistent sodium currents.^23^ These factors may together explain the lack of effect of carbamazepine on SDTC in both peripheral motor axons^41^ and cortical motor axons in the present study.

Differences between the medications may also stem from their interactions with different VGSC gating states. Carbamazepine binds preferentially to VGSCs in the fast inactivated state, delaying recovery from this state.^6^, ^47^, ^48^ Meanwhile, lacosamide enhances entry into the slow inactivated state and slows recovery from it.^6^, ^47^, ^48^ The latter observation aligns with our findings that lacosamide reduced the SDTC, as the persistent sodium currents involved are prone to slow inactivation,^51^ although we note that there is indirect evidence of carbamazepine affecting slowly inactivating sodium currents.^52^

Finally, carbamazepine may exert multiple actions that can interact with or mask its effect on VGSCs, such as its influence on GABAergic synaptic transmission. One TMS study reported effects on cortical silent periods mediated by GABA‐B receptors.^10^ However, these findings have not been consistently replicated,^11^ and the effects on GABAergic inhibition remain unexplored in the present study. Interestingly, it has been suggested that the strength–duration behavior of excitatory and inhibitory neurons may differ,^53^ which could complicate the evaluation of GABAergic inhibition in human cortex, as TMS intensities are typically set relative to motor threshold. One solution would be to simultaneously examine the strength–duration behavior of both excitatory and inhibitory synaptic inputs. This approach would benefit the study of novel sodium channel blockers that selectively target VGSC isoforms differentially expressed in excitatory and inhibitory neurons,^54^ by providing evidence of effective and selective targeting.

### 4.4 Directions for future research

This study confirmed the sensitivity of strength–duration metrics to sodium channel‐blocking medications. We selected carbamazepine because it is one of the most widely prescribed and well‐characterized sodium channel blockers, with established effects on TMS‐derived measures of cortical excitability.^10^, ^11^, ^12^ Although we acknowledge that carbamazepine may have off‐target actions, its use provided a strong benchmark for interpreting the sensitivity of our novel measures, particularly in comparison to lacosamide. Future work could assess the specificity of strength–duration metrics by testing drugs not expected to influence VGSCs, such as levetiracetam,^14^, ^20^ or by evaluating agents with potentially fewer off‐target effects, such as oxcarbazepine. This could help further delineate axonal versus synaptic contributions to cortical excitability.

Although our TMS‐electromyographic approach effectively probes axonal excitability in motor cortex, its spatial scope is limited. The development of equivalent TMS‐electroencephalographic (EEG) protocols could allow assessment of non‐motor regions, supporting the study of region‐specific pathophysiology and pharmacological responses, for example, in focal epilepsies. Prior work showing pulse width sensitivity of TMS‐evoked EEG supports this possibility.^55^

More broadly, strength–duration metrics may ultimately provide a valuable tool for predicting treatment response, monitoring efficacy, and guiding dose titration in a data‐driven, personalized manner. Future studies should evaluate the clinical utility of this approach.

## 5 CONCLUSIONS

Our findings highlight distinct effects of sodium channel‐blocking medications on strength–duration behavior in human motor cortex neurons. Lacosamide produced changes consistent with selective axonal sodium channel blockade, whereas carbamazepine showed more complex effects. These results underscore the value of strength–duration metrics for probing drug‐specific VGSC interactions and support their further development as mechanistic markers in human neurophysiology.

## AUTHOR CONTRIBUTIONS

**Lorenzo Rocchi:** Conception or design of the work; acquisition, analysis, or interpretation of data for the work; drafting the work or revising it critically for important intellectual content. **Kate Brown:** Acquisition, analysis or interpretation of data for the work; drafting the work or revising it critically for important intellectual content. **Alessandro Di Santo:** Acquisition, analysis, or interpretation of data for the work; drafting the work or revising it critically for important intellectual content. **Hannah Smith:** Acquisition, analysis, or interpretation of data for the work; drafting the work or revising it critically for important intellectual content. **Angel V. Peterchev:** Acquisition, analysis, or interpretation of data for the work; drafting the work or revising it critically for important intellectual content. **John C. Rothwell:** Conception or design of the work; acquisition, analysis, or interpretation of data for the work; drafting the work or revising it critically for important intellectual content. **Ricci Hannah:** Conception or design of the work; acquisition, analysis, or interpretation of data for the work; drafting the work or revising it critically for important intellectual content. All authors approved the final version of the manuscript, and all agree to be accountable for all aspects of the work in ensuring that questions related to the accuracy or integrity of any part of the work are appropriately investigated and resolved. All persons designated as authors qualify for authorship, and all those who qualify for authorship are listed.

## FUNDING INFORMATION

R.H.'s contribution was supported by the Academy of Medical Sciences Springboard scheme (SBF009/1073), which is funded by British Heart Foundation, Diabetes UK, the Government Department for Science, Innovation and Technology, and Wellcome. A.V.P.'s contribution to the reported research was supported by the National Institutes of Health under award numbers RF1MH124943, R01NS117405, and R01MH128422. The content is solely the responsibility of the authors and does not necessarily represent the official views of the funding agencies.

## CONFLICT OF INTEREST STATEMENT

A.V.P. is an inventor on patents on TMS technology and has received consulting fees and patent royalties for a license on the cTMS (Elevate TMS) technology used in this study from Rogue Research; equity options, scientific advisory board membership, and consulting fees from Ampa Health; consulting fees from Magnetic Tides and Soterix Medical; equipment loan from MagVenture; and research funding from Motif. The other authors have no relevant disclosures. We confirm that we have read the Journal's position on issues involved in ethical publication and affirm that this report is consistent with those guidelines.

## Supporting information


Appendix S1.

## Data Availability

All data and code used for analysis and figure generation are freely accessible at the following link: https://osf.io/d6vf2/.
